# Synthesis, properties and surface self-assembly of a pentanuclear cluster based on the new π-conjugated TTF-triazole ligand

**DOI:** 10.1038/srep25544

**Published:** 2016-05-06

**Authors:** Long Cui, Yan-Fang Geng, Chanel F. Leong, Qian Ma, Deanna M. D’Alessandro, Ke Deng, Qing-Dao Zeng, Jing-Lin Zuo

**Affiliations:** 1 State Key Laboratory of Coordination Chemistry, School of Chemistry and Chemical Engineering, Collaborative Innovation Center of Advanced Microstructures, Nanjing University, Nanjing, 210093, P.R. China; 2 CAS Key Laboratory of Standardization and Measurement for Nanotechnology, CAS Center for Excellence in Nanoscience, National Center for Nanoscience and Technology, Beijing, 100190, P.R. China; 3 School of Chemistry, The University of Sydney, New South Wales 2006, Australia

## Abstract

The new π-extended redox-active ligand with both TTF and triazole units, 6-(4,5-bis(propylthio)-1,3-dithiol-2-ylidene)-1H-[1,3]dithiolo[4′,5′:4,5]benzo [1,2-d] [1–3]triazole, has been successfully prepared. Based on the versatile ligand and Cu(tta)_2_ precursors (tta^−^ = 4,4,4-trifluoro-1-(thiophen-2-yl)butane-1,3-dione), a TTF-based pentanuclear Cu^II^ cluster (Cu_5_(tta)_4_(TTFN_3_)_6_) is synthesized and structurally characterized. Their absorption and electrochemical properties are investigated. Antiferromagnetic couplings are operative between metal ion centers bridged by triazoles in the complex. The self-assembled structure of the cluster complex on a highly oriented pyrolytic graphite (HOPG) surface was observed using scanning tunneling microscopy and density functional theory (DFT) calculations have been performed to provide insight into the formation mechanism. The introduction of the redox-active TTF unit into the cluster complexes with interesting magnetic properties renders them promising candidates for new multifunctional materials.

Over the last decade, the elaboration of molecular-based materials involving an interplay and synergy between multiple physical properties remains an exciting and complex challenge for scientists^1^,^2^,^3^,^4^. In particular, materials which possess electrical and magnetic properties have received significant attention due to their important applications in molecular spintronics^5^,^6^. To obtain such multi-property systems, some organic ligands are particularly suitable due to their electroactivity and strong electron delocalization. Among them, tetrathiafulvalene (TTF) and its derivatives which have strongly electron-donating and attractive reversible redox properties, have been successfully employed in the preparation of functional materials^7^,^8^,^9^. Controlled assembly of these functional molecules on surfaces with desirable dimensions and morphologies has become very attractive for chemists and material scientists.

To obtain a synergy between these two physical properties, a strong coupling between the localized d electrons and the mobile π electrons must be established^10^. A popular strategy to achieve an enhancement of π -d interactions in such dual-property materials is the direct coordination of paramagnetic metal ions to the TTF core through coordinating groups^11^,^12^,^13^,^14^,^15^,^16^,^17^. In recent years, various TTF derivatives which have metal-ion binding groups have been designed and synthesized for multifunctional materials. Although nitrogen heterocycles, such as pyridines, bipyridines, terpyridines and pyrazoles amongst others^18^,^19^,^20^,^21^,^22^,^23^,^24^,^25^,^26^, have been widely employed in such electroactive ligands and complexes, triazoles have received relatively limited attention^27^. Triazole heterocycles have proven to be efficient ligands for spin-crossover (SCO) Fe^II^ complexes by virtue of their ligand-field strengths which are favorable for SCO^28^,^29^. In particular, Fe^II^ one-dimensional triazole-based materials show promise in information technology applications^30^,^31^,^32^,^33^. From a synthetic point of view, 1,2,3-triazoles offer the possibility of several coordination sites and possess a rich variety of binding and bridging modes. Hence, they are expected to be versatile building blocks for the preparation of coordination materials.

Recently, Avarvari and co-workers reported a new TTF-triazole ligand with 1-substituted benzyl (TTF moieties are linked to the triazole through an C-C single bond) and its Cu^II^ complex^34^. However, to further enhance the interaction of conducting and magnetic π -d systems, it is important to shorten the distance between the magnetic metal ion and the conducting TTF units by designing new π -conjugated ligands.

Herein, a new π -extended redox-active ligand with both TTF and triazole units, (6-(4,5-bis(propylthio)-1,3-dithiol-2-ylidene)-1H-[1,3]dithiolo[4′ ,5′ :4,5]benzo[1,2-d][1–3]triazole) (**L**, Fig. 1), has been successfully prepared for the first time. Reaction of **L** with Cu(tta)_2_ afforded a pentanuclear Cu^II^ cluster complex **1** (Cu_5_(tta)_4_(TTFN_3_)_6_). All structures were characterized by single-crystal X-ray diffraction, solution state electrochemistry, UV–vis absorption spectroscopy and solution state UV–vis spectroelectrochemistry. The magnetic properties and self-assembled structure on highly oriented pyrolytic graphite (HOPG) surface of **1** were also investigated.

## Results and Discussion

### Structural description

The solid-state structures of **L** and **1** were determined by single-crystal X-ray diffraction. The structure of the ligand **L** with its atomic numbering scheme is shown in Fig. S1. The ligand **L** crystallizes in the triclinic crystal system, space group *P*ī. The fused ring system is approximately planar, but the propyl groups stretch out of the plane. The central C7− C8 bond length of 1.331(8) Å is in agreement with the neutral state of the donor^35^,^36^. Intermolecular N···H hydrogen bonds are observed for the supramolecular structure (Fig. S2, SI).

**Figure 2:**
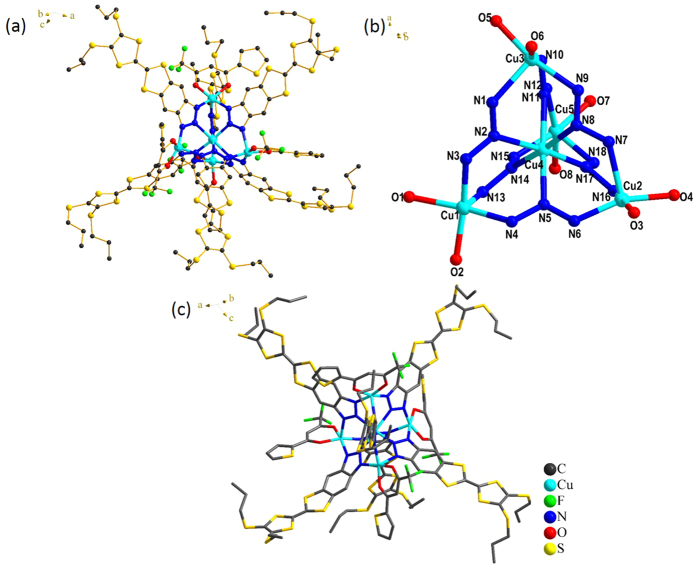
(**a**) Crystal structure of complex **1**. (**b**) Perspective view of the molecular structure of 1 showing the atom labels. For clarity, all the fluorine atoms and thiophene units of tta^−^ and the hydrogen atoms of TTFN_3_ were omitted. (**c**) Top view of complex **1**. The arrangement of six ligands in an octahedral geometry comprises of the equatorial plane occupied by four ligands and the two axial positions occupied by the other two.

Single-crystal X-ray diffraction analysis revealed that complex **1** crystallizes in the monoclinic space group *C*2/c. As shown in Fig. 2, the pentanulear Cu^II^ cluster consists of a distorted tetrahedral arrangement of four five-coordinate Cu^II^ ions centered about the fifth one. Each of the six *μ*_3_-TTFN_3_ ligands straddles an edge of the tetrahedron and is bound to the central metal through the nitrogen atom in the 2-position. Six nitrogen atoms from six TTFN_3_ ligands form the coordination sphere of the central Cu^II^ ion, while each Cu^II^ ion on the apical positions is coordinated by three nitrogen atoms from three TTFN_3_ ligands and two oxygen atoms from a bidentate *β*-diketonate ligand. The central Cu^II^ ion adopts a distorted octahedral coordination geometry with average Cu-N distances of 2.108(3) Å. The apical Cu^II^ ion is in an approximately square-based pyramidal environment. Two TTFN_3_ nitrogen atoms and two oxygen atoms of the bidentate donor molecule are on the square, whilst the third TTFN_3_ nitrogen atom lies on the apical position. For the Cu^II^ bridged by axial TTFN_3_ anions, the average distance between the apical Cu^II^ ions is 5.965(6) Å, while the average distance between the central and the apical Cu^II^ ions is 3.654(3) Å.

### Spectroscopic properties

The absorption spectra of compounds **L** and **1** in CH_2_Cl_2_ at room temperature were measured (Fig. S3, SI). The spectrum of **L** is characterized by strong absorption bands at high energy (λ  <  340 nm) that can be assigned to the intraligand (IL) π –π * transition, and a weak absorption band (340–470 nm) at lower energy that can be assigned to IL charge transfer (ILCT) from the highest occupied molecular orbital in TTF to the lowest unoccupied molecular orbital in the electron-accepting benzotriazole unit. Metalation of the free ligand leads to a red shift of charge-transfer transitions and to additional metal to ligand charge-transfer (MLCT) bands in the range of 340–380 nm^37^.

### Electrochemical properties

Both cyclic voltammetry (CV) and square wave (SW) measurements of compounds **L** and **1** were conducted in 0.1 M [*n*-Bu_4_N]PF_6_ in CH_2_Cl_2_ electrolyte. Redox potentials were referenced to the ferrocenium/ ferrocene couple (Fc^+^/Fc). Cyclic and square wave voltammetry measurements on the compound **L** revealed two reversible one-electron oxidation processes at *E*_*1/2*_ =  0.175 and 0.575 V *vs.* Fc^+^/Fc (Figs S4 and S5, SI) which can be attributed to the oxidation of the neutral TTF core to its radical cation species, followed by a further oxidation to its dication state, respectively. These two oxidation processes persist in complex **1**, and occur at similar *E*_1/2_ values of 0.176 and 0.592 V. Owing to the extended aromatic bridge between the coordinating triazole N-donor and the TTF unit, it is explicable that coordination to Cu^II^ does not significantly influence the oxidation potential of the TTF core. This suggests that there is a lack of significant coupling between the TTF cores and the metal cluster^38^,^39^,^40^. The cyclic voltammetric data on **L** and **1** are summarized and collected in Table S4. The oxidation potentials associated with TTF^•+^/TTF and TTF^2+^/TTF^•+^ seen in **L** and **1** are in agreement with TTF derivatives with larger conjugated π -systems^39^,^41^.

Additional features arise in the electrochemical data for complex **1** which are not present in that of the ligand alone. For example, a quasi-reversible reduction wave found at an *E*_*1/2*_ value of − 1.360 V for complex **1**, and is tentatively assigned to the Cu^II/I^ redox couple (Fig. S6, SI). Upon redox switching of the Cu^II^ centre to Cu^I^, a new irreversible oxidation peak at an onset potential of − 0.080 V was observed upon the reverse scan in the anodic region; this process was better exemplified by SW voltammetry revealing a broad process as a shoulder at − 0.080 V.

Integration of the peaks in the SW voltammogram of **1**, shows that the ratio of peaks for the Cu^II/I^, TTF/TTF^+^, and TTF^+^/TTF^2+^ couples is 1:8:7. This ratio is close to 1:6:6 which suggests the reduction of only one Cu^II^ ion in **1**, compared to the oxidation of 6 **L** ligands. As the SW measurement was collected over the range − 1.92 to 1.08 V versus Fc^+^/Fc, degradation of the complex due to the irreversible Cu^II/I^ couple is likely to account for the discrepancy between the observed and expected ratios.

### Solution State Spectroelectrochemistry

**Figure 3:**
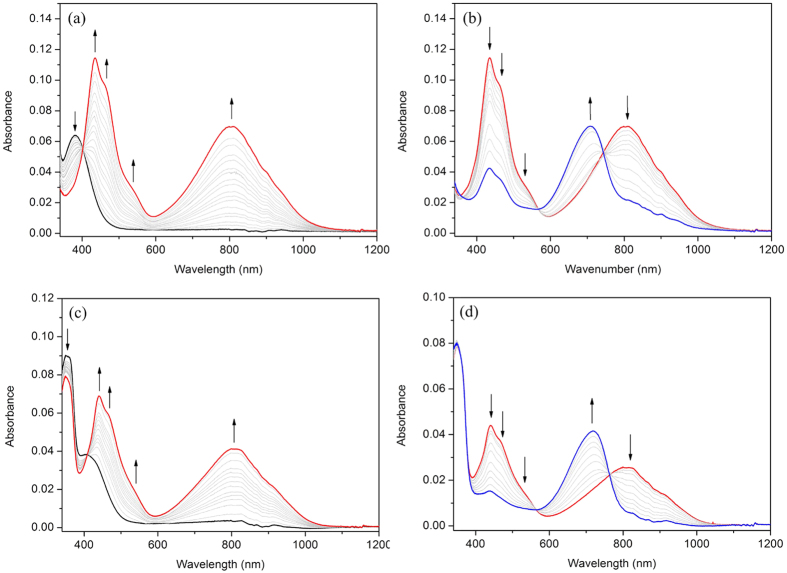
Solution state spectroelectrochemistry (SEC) of L and 1. The spectral progressions show the oxidation of the neutral compounds (black) to their radical cation (red) and dication (blue) states. These states were obtained at applied potentials (vs Fc^+^/Fc) of (**a**) 0.19 V and (**b**) 0.54 V for **L**, and at (**c**) 0.20 V and (**d**) 0.50 V for **1**. Measurements were performed in 0.1 M [*n*-Bu_4_N]PF_6_ in CH_2_Cl_2_ and the arrows indicate the direction of spectral change. Grey curves represent the system in transition from one species to another at time intervals of 2.3 minutes.

As shown in Fig. 3a, the ligand was oxidized to its radical cation at 0.19 V, which resulted in the lowering of intensity of the ILCT band at 380 nm and the formation of new, lower energy bands at 430, 460, 540 and 800 nm. These spectral changes are in agreement with those observed previously in related compounds^42^,^43^. To further confirm the optical signature of the radical cation, DFT calculations were performed (Figs S7 and S8 and Table S5, SI). The calculated absorption spectra are in favorable agreement with those obtained experimentally. The lowest energy excited state of the radical corresponds to a HOMO(β ) →  LUMO(β ) transition, with the β -HOMO delocalized over both subunits of the radical cation. This corresponds to transitions of mixed character and π π * transitions that are localized on the triazole unit.

Further oxidation of the TTF radical cation at 0.54 V led to formation of the dication species which corresponded to a decrease in intensity of the bands at 430, 460, 540 and 800 nm and the appearance of a new band at 700 nm. The ligand however, was unstable to complete oxidation to its dication state, and hence equilibrium was not achieved in Fig. 3b (blue spectrum).

The complex **1**, exhibited similar behavior to that of **L**, suggesting that the redox properties of the Cu^II^ derivative are localized on the TTF-based ligand (Fig. 3c,d). At 0.20 V, new bands appeared at 440, 460, 540 and 810 nm, whilst a decrease in intensity was observed in the MLCT band at 350 nm− changes which correspond to the formation of the TTF radical cation. At a more oxidizing potential of 0.50 V, the bands at 440, 460, 540 and 810 nm diminished as a new band appeared at 720 nm, as the dication TTF species was formed. As noted for the ligand, compound **1** was unstable to complete oxidation to its dication state, and hence equilibrium was not achieved in Fig. 3d (blue spectrum).

### Magnetic properties

**Figure 4:**
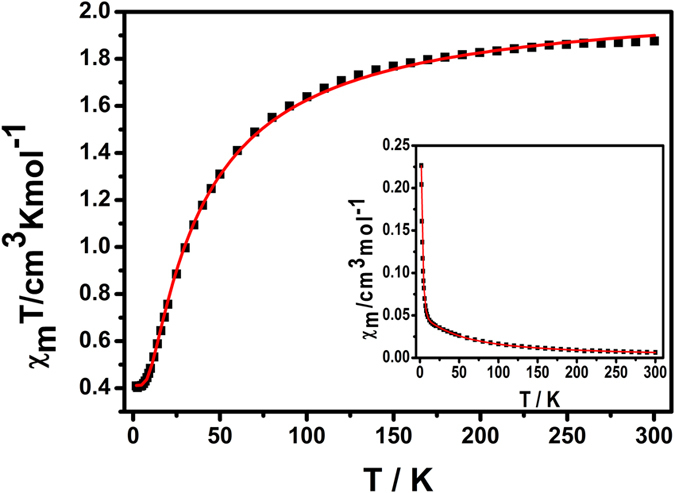
Temperature dependence of the χ_M_*T* product for **1** at 1000 Oe. The red solid line represents the best fit to the data. Inset: Temperature dependence of the χ _M_ product. The red solid line represents the best fit to the data.

As illustrated in Fig. 4, direct current (DC) magnetic susceptibility measurements for **1** were collected in the temperature range of 1.8–300 K at 1000 Oe. The χ _M_*T* value of 1.88 cm^3^·K·mol^−1^ at 300 K is close to the theoretical one (1.875 cm^3^·K·mol^−1^) calculated from five isolated Cu^II^ (*S*_Cu_ =  1/2) spins with *g* =  2. Upon cooling, the *χ*_M_*T* value gradually decreases followed by an abrupt decline, indicating the antiferromagnetic coupling (AF) between neighboring Cu^II^ ions. The magnetic susceptibility above 50 K obeys the Curie-Weiss law with *C* =  2.05(1) cm^3^·K·mol^−1^ and a Weiss constant *θ* =  **−**24.96(9) K (Fig. S9, SI), suggesting the presence of a dominant antiferromagnetic coupling between Cu^II^ ions. The field dependence of magnetization was measured at 1.8 K (Fig. S10, SI). The magnetization increases slowly to a maximum value of 0.98 *Nβ* at 70 kOe which is close to the expected result of 1 *Nβ.* To probe the underlying magnetic exchange characteristics, a highly symmetric model (Fig. S11, SI)^44^ was employed. The best fit to the data for the compound between 1.8 and 300 K gave *g* =  2.09(1), *J*_1_ =  **−**8.34(1) cm^−1^, *J*_2_ =  **−**8.34(1) cm^−1^ (*R* =  1.24 ×  10^−4^), showing antiferromagnetic interactions between Cu^II^ ions through the triazole bridges.

### Scanning tunneling microscopy (STM) investigations

The surface adsorption and the corresponding self-assembly of ligand-metal complexes have been the subject of significant investigation. This technique can provide a fundamental understanding of the coordinated interaction of molecules on a surface, as well as an insight into the potential surface applications^45^. Surface coordination networks based on Cu^II^ and organic ligands have emerged as a novel class of surface materials^46^,^47^,^48^. Herein, the assembly of complex **1** formed by ligand **L** and Cu^II^ was investigated by STM techniques under ambient conditions.

**Figure 5:**
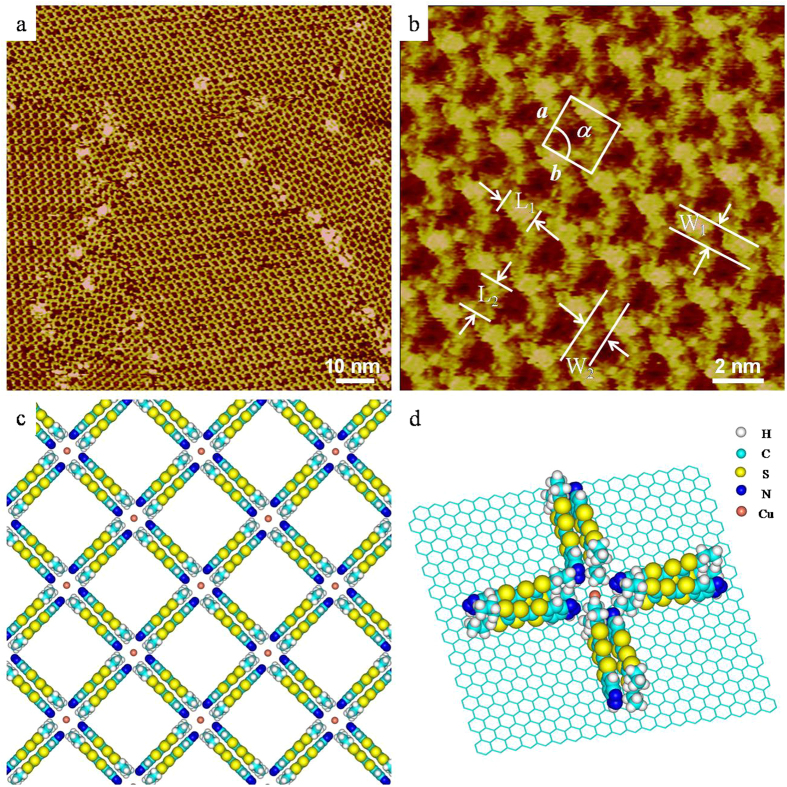
(**a**) Large scale and (**b**) high resolution STM images of Cu^II^ complex coordinated by ligand **L** on HOPG surface (*I*_set_ =  299.1 pA, *V*_bias_ =  699.8 mV). (**c**) A suggested molecular model. (**d**) A schematic arrangement of Cu^II^ complex on HOPG surface.

As shown in Fig. 5, complex **1** can spontaneously form relatively large area periodic assemblies on a HOPG surface. High-resolution STM images as shown in Fig. 5b clearly reveal a 15 nm ×  15 nm STM image of complex **1** on the HOPG, where the arrangement of complexes and the network structure is evident. The length L_1_ and L_2_ of the bright rectangles were measured to be 1.5 ± 0.1 nm, while the width W_1_ and W_2_ were estimated to be 0.9 ± 0.1 nm. A square unit cell is described as shown in Fig. 5b. The measured unit parameters are as follows: *a* =  *b* =  2.4 ±  0.1 nm, and α  =  90 ±  1°. As mentioned above, the length of ligand **L** is circa 1.5 nm, and the width of **L** is circa 0.5 nm. From the molecular size and shape, we assign each rectangle to two parallel **L** molecules. One Cu^II^ occupies the dark crossing region^49^,^50^,^51^. In particular, two adjacent **L** arrange in the opposite direction through π -π interactions forming a 2-dimensional array as shown in Fig. 5c. A stereospecific coordination structure is shown in Fig. 5d, which is in agreement with the single crystal results as illustrated in Fig. 2c. In the crystal structure, the central six-coordinate Cu^II^ labeled as Cu4 is connected to six nitrogen atoms marked as N2, N5, N8, N11, N14, N17 from six TTFN_3_ ligands. On the surface, four TTFN_3_ ligands make up a quadrangle. Additionally, the bright spots at the crossing points in Fig. 5a can be assigned to the complexes formed by Cu^II^ with five or six ligands out of the HOPG surface. Therefore, the adsorption geometry of the complex on a surface can offer important insight into the complex structure and intermolecular packing interactions.

To better illustrate the self-assembled architecture of the coordinated complex, simulations were performed by density functional theory (DFT) calculations. The calculated results showed that two adjacent **L** were oriented parallel to each other in opposite directions. The interaction energy is about − 30.06 kcal mol^−1^. Considering the chemical structure of **L**, we suggest that both π -π interactions between the π -conjugated rings and the S-S interaction between the adjacent **L** contribute to such strong interactions. In the crystal structure, the complexes formed by Cu^II^ should be coordinated to five or six ligands. However, it is interesting that on the HOPG surface, we only observed the regular assembly formed by quadrangles. This result implies that on the surface, each Cu^II^ is coordinated to four TTFN_3_ ligands. The coordination energy between Cu^II^ and the TTFN_3_ ligand is about − 11.06 kcal mol^−1^. We have optimized the assembly of coordinated Cu^II^ complexes on the surface, and the calculated molecular model for this assembly is shown in Fig. 5c. The calculated lattice parameters for 2D networks are *a* =  *b* =  2.4 nm, and α  =  90°, which agree well with the experimental data. With careful inspection of the results, we found that coordinated Cu^II^ complexes interact with the HOPG surface via a van der Waals interaction. The interaction between the complex and substrate is sufficiently strong (− 181.74 kcal mol^−1^) to support the formation of a regular assembly of coordinated Cu^II^ complexes on the HOPG surface.

In summary, the novel redox-active π -extended triazole ligand with TTF has been synthesized and thoroughly characterized. The versatile coordination ability for the ligand was demonstrated by the isolation of the interesting TTF-based pentanuclear Cu^II^ cluster (Cu_5_(tta)_4_(TTFN_3_)_6_) bearing six electroactive, functionalized TTF ligands. Each of the six μ _3_-TTFN_3_ ligands straddles an edge of the tetrahedron and is bound to the Cu^II^ ions through the nitrogen atom in the 2-position. All structures have been characterized by solution state electrochemistry, UV–vis absorption spectra and UV–vis spectroelectrochemistry. Magnetic studies show that antiferromagnetic couplings are operative between metal ion centers.

The self-assembled structure imaged by STM shows the arrangement of complex **1** on a HOPG surface. Owing to the intrinsic abilities of TTF moieties to form shorter S···S contacts and π -π stacking, complex **1** constitutes an interesting building block for the elaboration of magnetic conductor or semiconductor materials. DFT calculations have also been performed to reveal the formation mechanism. Further investigations of the TTFN_3_ ligand in Fe^II^ complexes may pave the way towards new spin-crossover, switchable materials with multi-stimuli responses. This work is currently underway in our laboratory.

## Methods

Details of the synthesis and characterization methods for ligand **L** and complex **1** are described in the Supplementary Information. The final crystallographic data and the values of *R*_1_ and w*R*_2_ are listed in Table S1. Selected bond distances and angles for ligand **L** and complex **1** are listed in Tables S2 and S3. CCDC-1059274 (**L**) and 1059275 (**1**) contain the supplementary crystallographic data for this paper.

STM imaging was performed on the monolayers of complex **1**, which was fabricated by depositing the EtOH solution with low concentration (less than 1.0 ×  10^−4^ mol/L) onto the HOPG surface, under ambient condition *via* Nanoscope Multimode SPM (Bruker Nano Inc.) with constant current mode.

Theoretical calculations for geometry optimizations and UV-Vis spectra were carried out with Gaussian09 programs^52^. DFT and time-dependent DFT (TD-DFT) with the three-parameter B3LYP hybrid functional were employed. Calculations were carried out using with a 6–31 +  G** basis set for all atoms.

Theoretical calculations for STM were performed using DFT provided by the DMol3 code^53^. We used periodic boundary conditions (PBC) to describe the 2D periodic structure on the graphite in this work. The Perdew and Wang parameterization^54^ of the local exchange correlation energy was applied in local spin density approximation (LSDA) to describe exchange and correlation. All-electron spin-unrestricted Kohn-Sham wave functions were expanded in a local atomic orbital basis. For the large system, the numerical basis set was applied. All calculations were all-electron ones, and performed with a medium mesh. The self-consistent field procedure was employed with a convergence criterion of 10^−5^ au on the energy and electron density. Combined with the experimental data, we have optimized the unit cell parameters and the geometry of the adsorbates in the unit cell. When the energy and density convergence criteria are reached, we could obtain the optimized parameters and the interaction energy between adsorbates.

To evaluate the interaction between the adsorbates and HOPG, we designed a model system. Since adsorption on graphite and graphene should be very similar, we have performed our calculations on infinite graphene monolayers using PBC. In the superlattice, graphene layers were separated by 40 Å in the normal direction and represented by orthorhombic unit cells containing two carbon atoms. When modelling the adsorbates on graphene, we used graphene super cells and sampled the Brillouin zone by a 1 ×  1 ×  1 k-point mesh. The interaction energy *E*_inter_ of adsorbates with graphite is given by *E*_inter_ =  *E*_tot_(adsorbates/graphene) −  *E*_tot_(isolated adsorbates in vacuum) −  *E*_tot_(graphene).

## Additional Information

**How to cite this article**: Cui, L. *et al.* Synthesis, properties and surface self-assembly of a pentanuclear cluster based on the new π-conjugated TTF-triazole ligand. *Sci. Rep.* **6**, 25544; doi: 10.1038/srep25544 (2016).

## Supplementary Material

Supplementary Information

## Figures and Tables

**Figure 1:**
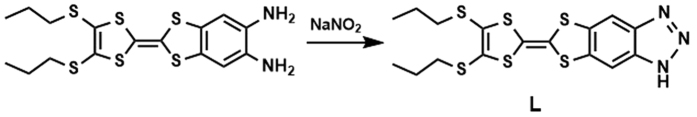
Synthesis of ligand L.
