# Supplementary material for: Synthesis, properties and surface self-assembly of a pentanuclear cluster based on the new π-conjugated TTF-triazole ligand
Source: Sci Rep. 2016 May 6;6:25544. doi: 10.1038/srep25544 (PMC4858759; doi:10.1038/srep25544)
Supplement: Supplementary Information [file srep25544-s1.doc]

Supplementary information for

Synthesis, properties and surface self-assembly of a pentanuclear cluster based on the new π-conjugated TTF-triazole ligand

Long Cui^1^, Yan-Fang Geng^2^, Chanel F. Leong^3^, Qian Ma^1^, Deanna M. D'Alessandro^3^, Ke Deng^2*^, Qing-Dao Zeng^2*^ & Jing-Lin Zuo^1*^

^1^State Key Laboratory of Coordination Chemistry, School of Chemistry and Chemical Engineering, Collaborative Innovation Center of Advanced Microstructures, Nanjing University, Nanjing, 210093, P.R. China

^2^CAS Key Laboratory of Standardization and Measurement for Nanotechnology, CAS Center for Excellence in Nanoscience, National Center for Nanoscience and Technology, Beijing, 100190, P.R. China.

^3^School of Chemistry, The University of Sydney, New South Wales 2006, Australia.

* Corresponding authors. E-mail: zuojl@nju.edu.cn; [zengqd@nanoctr.cn](mailto:zengqd@nanoctr.cn); kdeng@nanoctr.cn

**General Information.** All the reagents were commercially available and used without further purification. 5,6-Diamino-2-(4,5-bis(propylthio)-1,3-dithio-2-ylidene)-benzo[d]-1,3-dithiole was prepared according to the literature method.^1^ Elemental analyses for C, H and N were performed on the Elementar Vario MICRO analyzer. ^1^H NMR spectra were recorded on a Bruker DPX 400 MHz spectrometer with internal standard tetramethylsilane (TMS) and solvent signals as internal references at room temperature. Mass spectra were recorded on a Bruker Autoﬂex ^II^TM instrument for MALDI-TOF-MS. Absorption spectra were measured on a Shimadzu UV-3100 spectrophotometer. Solution state electrochemical measurements were carried out in a three-electrode cell employing a glassy-carbon working electrode, Pt wire auxiliary electrode and Ag wire reference electrode. Both cyclic voltammetry (CV) and square wave (SW) measurements were conducted in 0.1 M [*n*-Bu_4_N]PF_6_ in CH_2_Cl_2_ electrolyte degassed under Ar. Redox potentials were referenced to the ferrocenium/ferrocene couple (Fc^+^/Fc). Square wave voltammetry data were performed with a 2 mV step size, an amplitude of 80 mV and frequency of 19 Hz. Solution state UV–vis spectroelectrochemical data were collected on a CARY 5000 Spectrophotometer over the range 340-1200 nm. The electrochemical experiments were performed in a three-electrode quartz cell with a fused 0.6 mm quartz cuvette, and employing a Pt gauze working electrode, Pt wire auxiliary electrode and a Ag/Ag^+^ quasi-reference electrode in 0.1 M [*n*-Bu_4_N]PF_6_ in CH_2_Cl_2_ electrolyte. Spectra were collected at a given potential at a time interval of 2.3 minutes. When equilibrium was attained at a given potential (as evidenced by the superposition of two successive spectral traces), the potential was increased by a small increment (0.05 V) as the spectra continued to be monitored. At the point where no further changes in the spectrum were observed with a further small change in the potential (or where the isosbestic points began to deviate), the steady state spectrum for a given redox state of the ligand or compound had been attained. Potentials were applied using an eDAQ e-corder 410 potentiostat. The crystal structures were determined with a Siemens (Bruker) SMART CCD diffractometer. The cell parameters were retrieved using SMART software and refined by using SAINT^2^ for all observed reflections. The highly redundant data sets were reduced using SAINT and corrected both for Lorentz and polarization effects. The absorption corrections were applied using SADABS^3^ supplied by Bruker. The structures were solved by direct methods using the program SHELXL-97.^4^ The positions of metal atoms and their first coordination spheres were located from direct methods E-maps. The other non-hydrogen atoms were found in alternating difference Fourier syntheses and least-squares refinement cycles, and during the final cycles, refined anisotropically. Hydrogen atoms were placed in calculated positions and refined as riding atoms with a uniform value of *U*_iso_. Magnetic susceptibilities for polycrystalline samples were measured with the use of a Quantum Design MPMS-SQUID-VSM magnetometer in the temperature range 1.9-300 K. Field dependences of magnetization were measured using a Quantum Design MPMS-SQUID-VSM system in an applied field up to 70 kOe. Diamagnetic corrections were calculated using Pascal’s constants and an experimental correction for the diamagnetic sample holder was applied.

**Synthesis of L.** To a stirred solution of 5,6-diamino-2-(4,5-bis(propylthio)-1,3-dithio -2-ylidene)-benzo[d]-1,3-dithiole (432 mg, 1 mmol) in MeOH/THF (1:1, v/v, 30 mL) was added CH_3_COOH (1.2 mL) and NaNO_2_ (217 mg, 3 mmol) in H_2_O (5 mL) at 0 ^o^C. The reaction mixture was stirred overnight. Then the solvent was evaporated, and the residue was extracted with CH_2_Cl_2_ and washed with saturated NaHCO_3_ solution. The organic extract was purified by column chromatography (CH_2_Cl_2_/EtOAc) on silica gel to obtain **1** as a yellow solid. Yellow crystals suitable for the single-crystal X-ray analysis were obtained by slow evaporation of the CH_2_Cl_2_/CH_3_CN (2:1) solution of **1.** Yield: 297 mg (67%). Anal. Calcd for C_16_H_17_N_3_S_6_: C, 43.31; H, 3.96; N, 9.47. Found: C, 43.46; H, 4.26; N, 9.34; ^1^H NMR (400 MHz, CD_2_Cl_2_, ppm): δ=0.93 (t, J ~ 7.3 Hz, 6 H), 1.65~1.53 (m, 4 H), 2.74 (t, J ~ 7.2 Hz, 4 H), 7.67 (s, 2 H), 12.15 (s, 1 H); m/z (MALDI-TOF): 443.291 (M ^+^).

**Synthesis of complex 1.** A solution of TTF-triazole (43 mg, 0.1mmol) in CH_2_Cl_2_ (5 mL) was added to another solution of Cu(tta)_2_ (51 mg, 0.1 mmol) in CH_2_Cl_2_ (5 mL). The resulting deep green solution was stirred for 1 h at room temperature. The dark-green single crystal suitable for X-ray analysis was obtained by careful layering of a concentrated CH_2_Cl_2_ solution with a co-solvent EtOH/CH_2_Cl_2_ (1:1, v/v). Yield: 33%. Anal. Calcd for C_128_H_112_Cu_5_F_12_N_18_O_8_S_40_: C, 39.84; H, 2.93; N, 6.53. Found: C, 40.03; H, 3.11; N, 6.37.

**Figure S1** Molecular structure of **L** in front (a) and side (b) views.

**
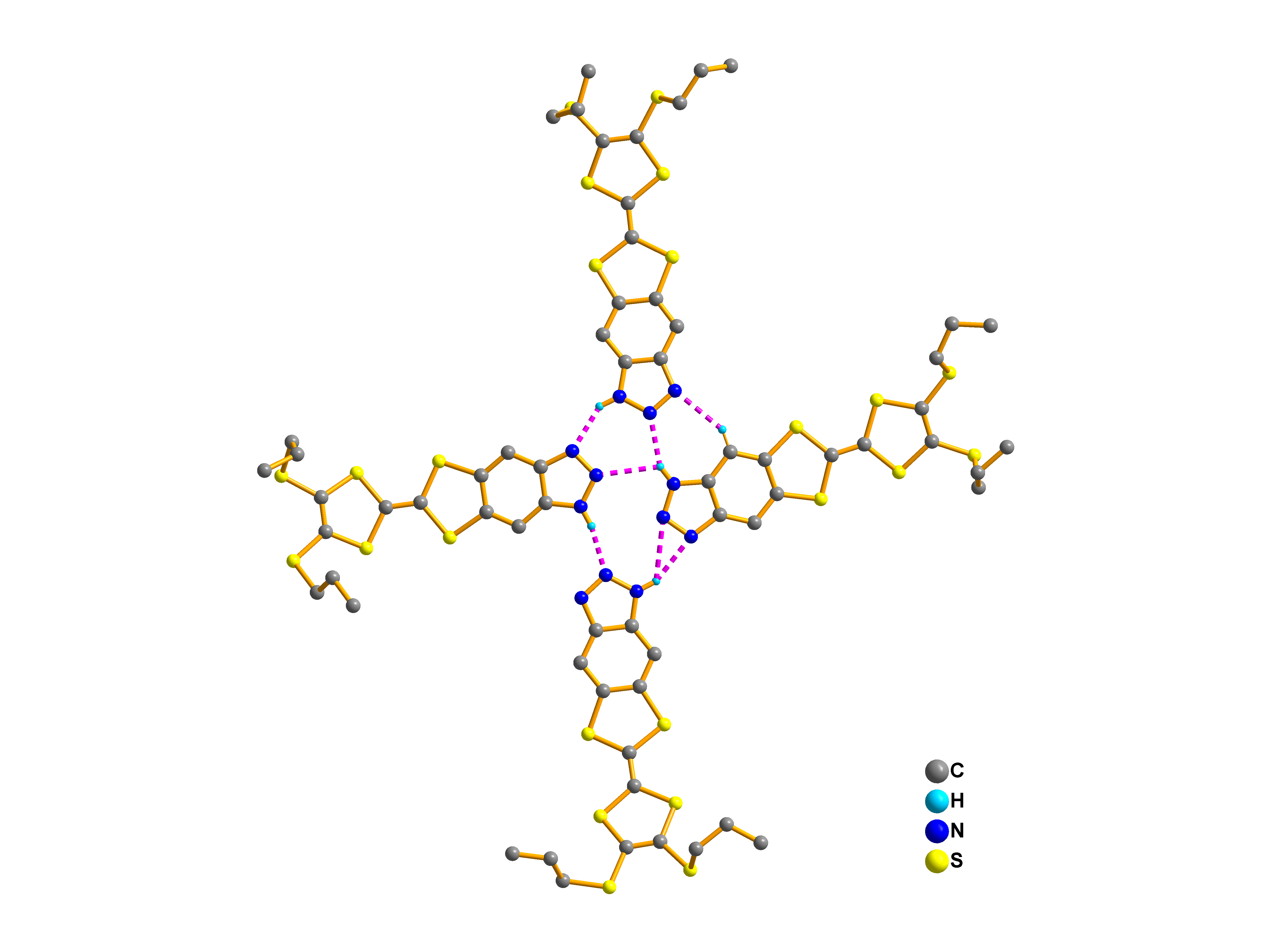
**

**Figure S2** Perspective view with an emphasis on the N···H hydrogen bond


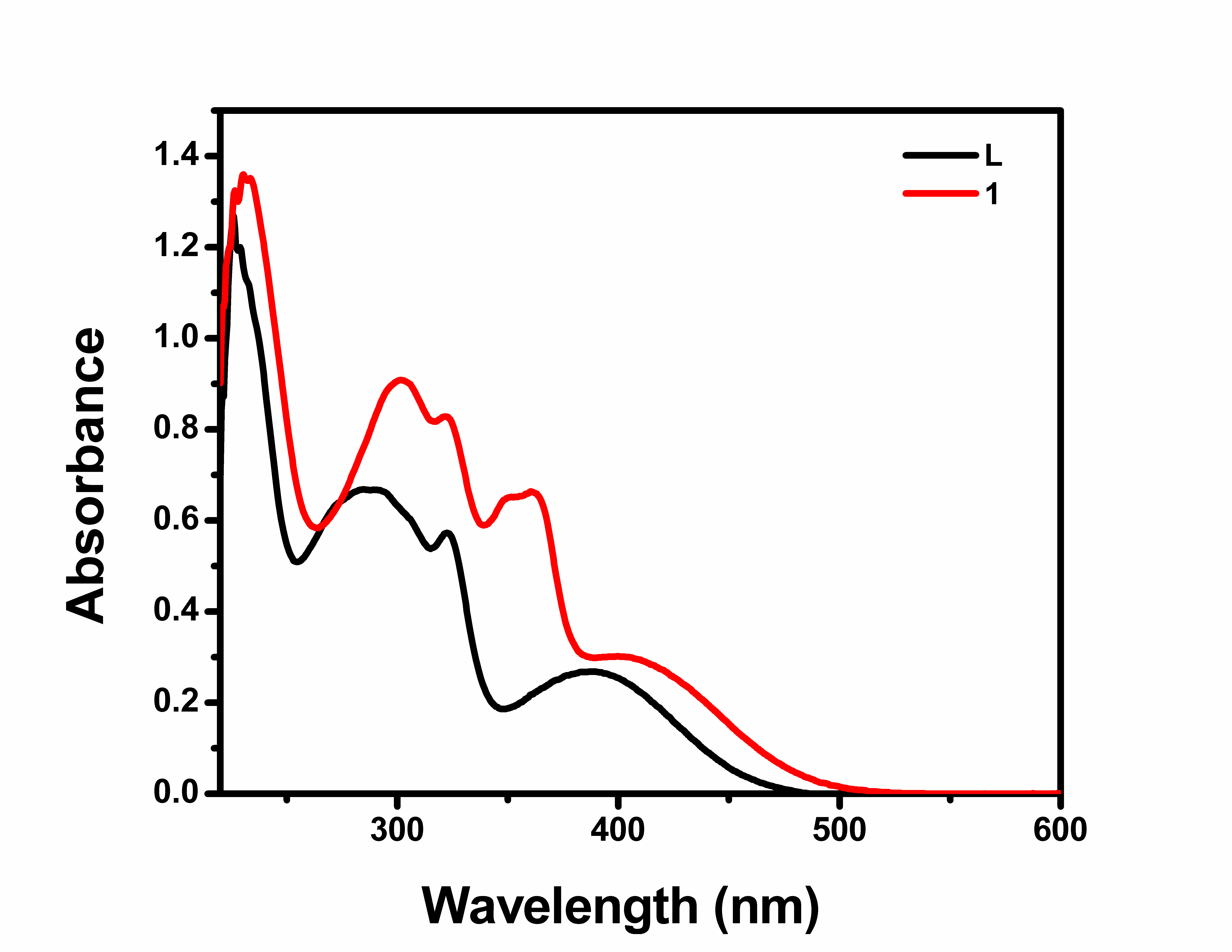


**Figure S3** UV–vis absorption spectra of compounds **L** and **1** in CH_2_Cl_2_ solution.


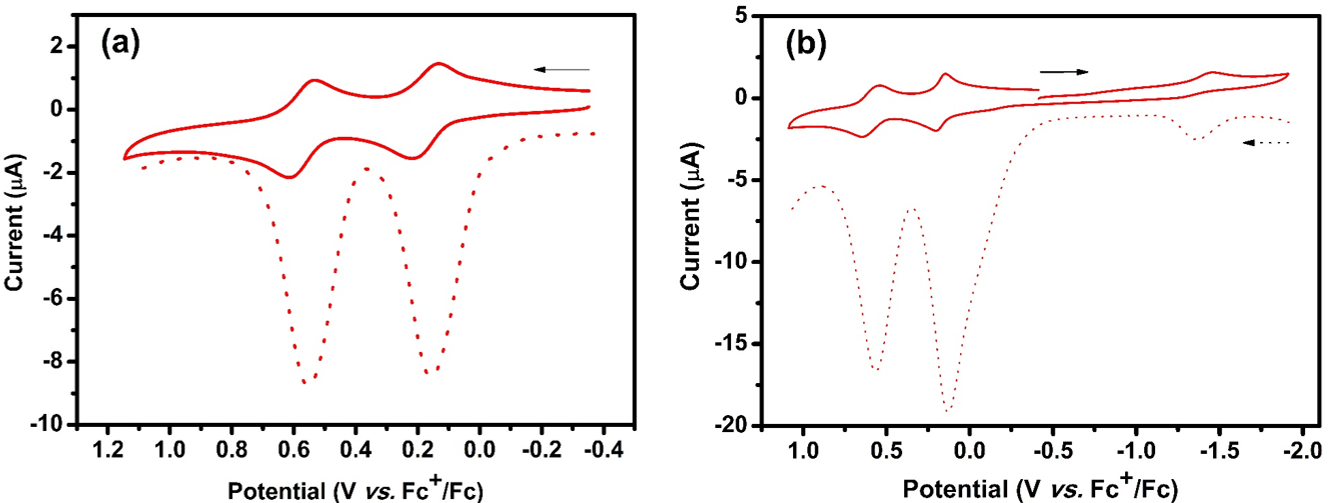


**Figure S4** Overlay of cyclic voltammetry (solid line) and square wave voltammetry (broken line) data for ligand **L** (a) and complex **1** (b). Measurements performed in 0.1 M [*n*-Bu_4_N]PF_6_ in CH_2_Cl_2_ (vs Fc^+^/Fc) and CVs run at 100 mV/s. Arrows indicate the direction of initial scan.


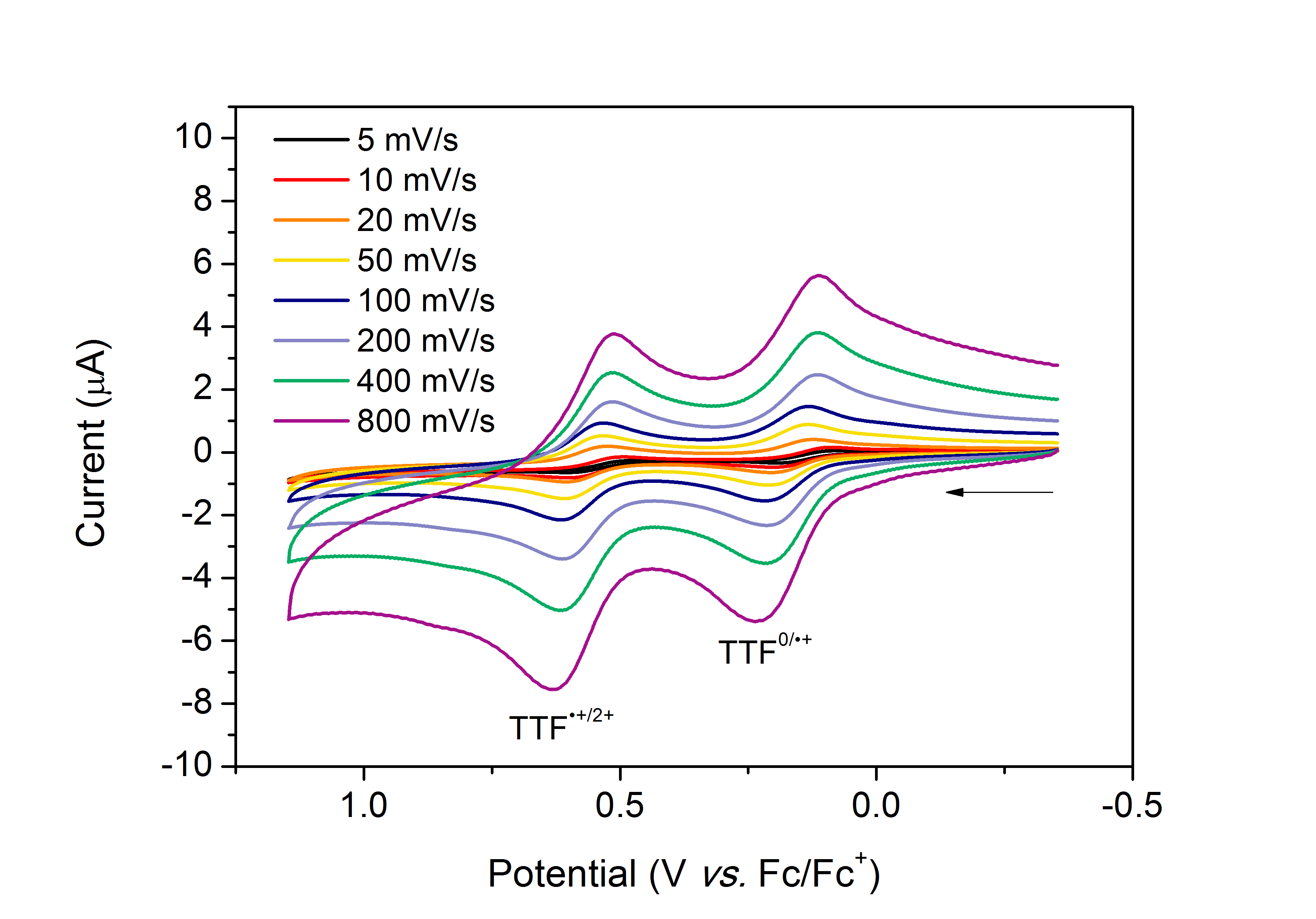


**Figure S5** Scan rate dependence CVs for L over multiple scan rates, demonstrating the reversibility of the redox processes.


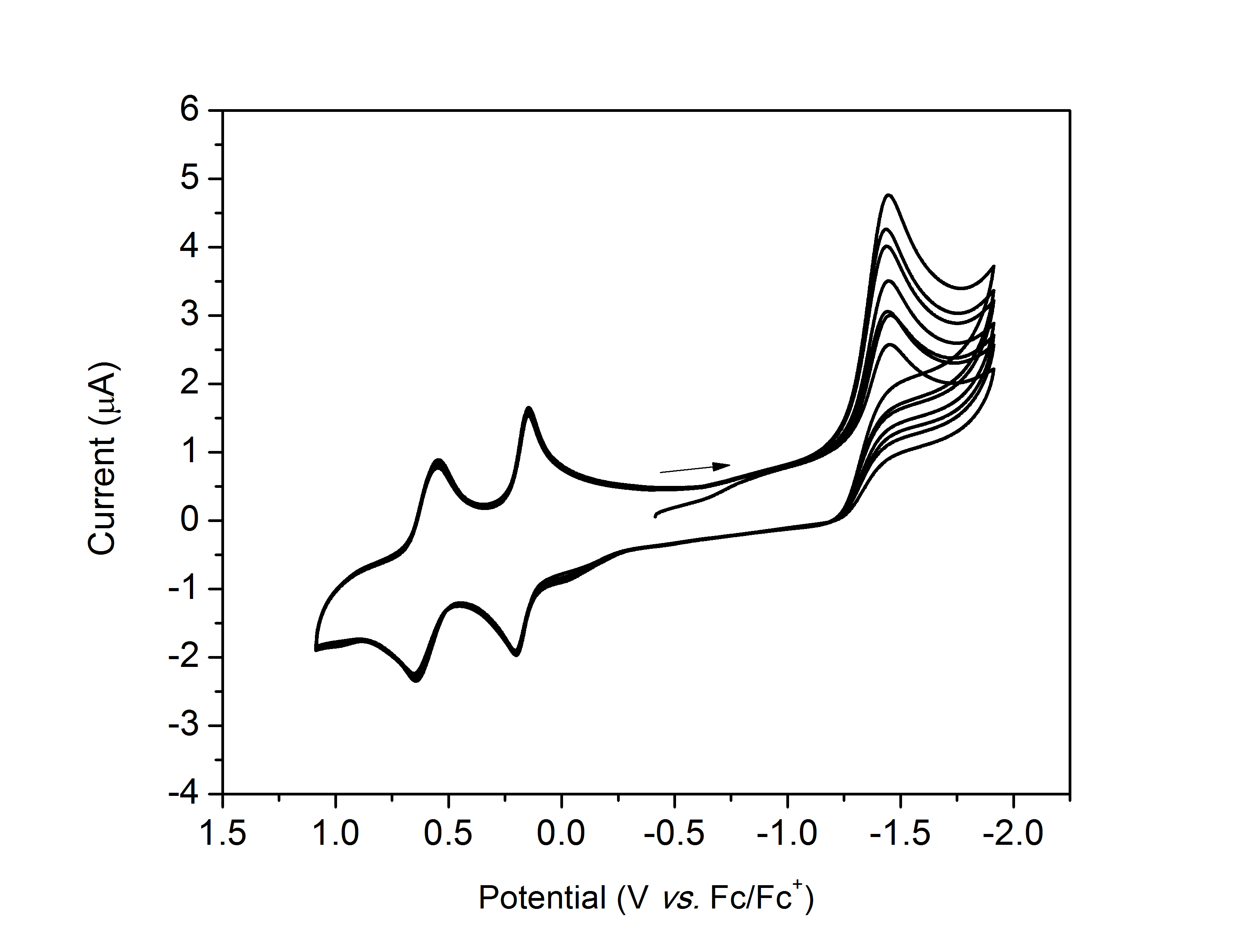


**Figure S6** CVs of 1 at 100 mV/s showing multiple cycles. The TTF-based oxidation processes are reproducible over the multiple cycles, while the reduction peak varies, supporting the irreversible nature of this process in the complex.


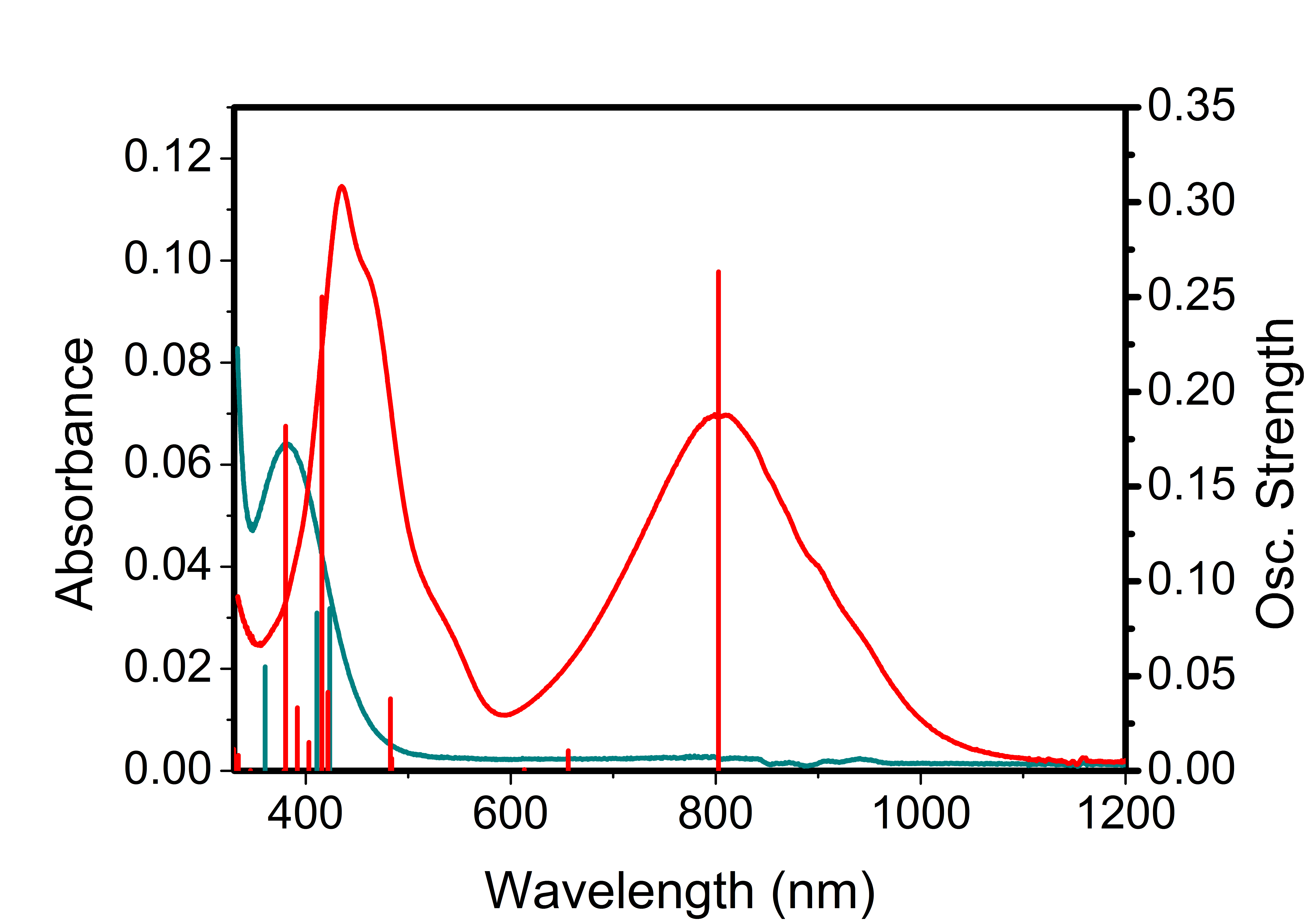


**Figure S7** Comparison of the experiment (line graph) and calculated absorption spectra (stick plot) of neutral (dark cyan) and one-electron-oxidized ligand **L** (red).


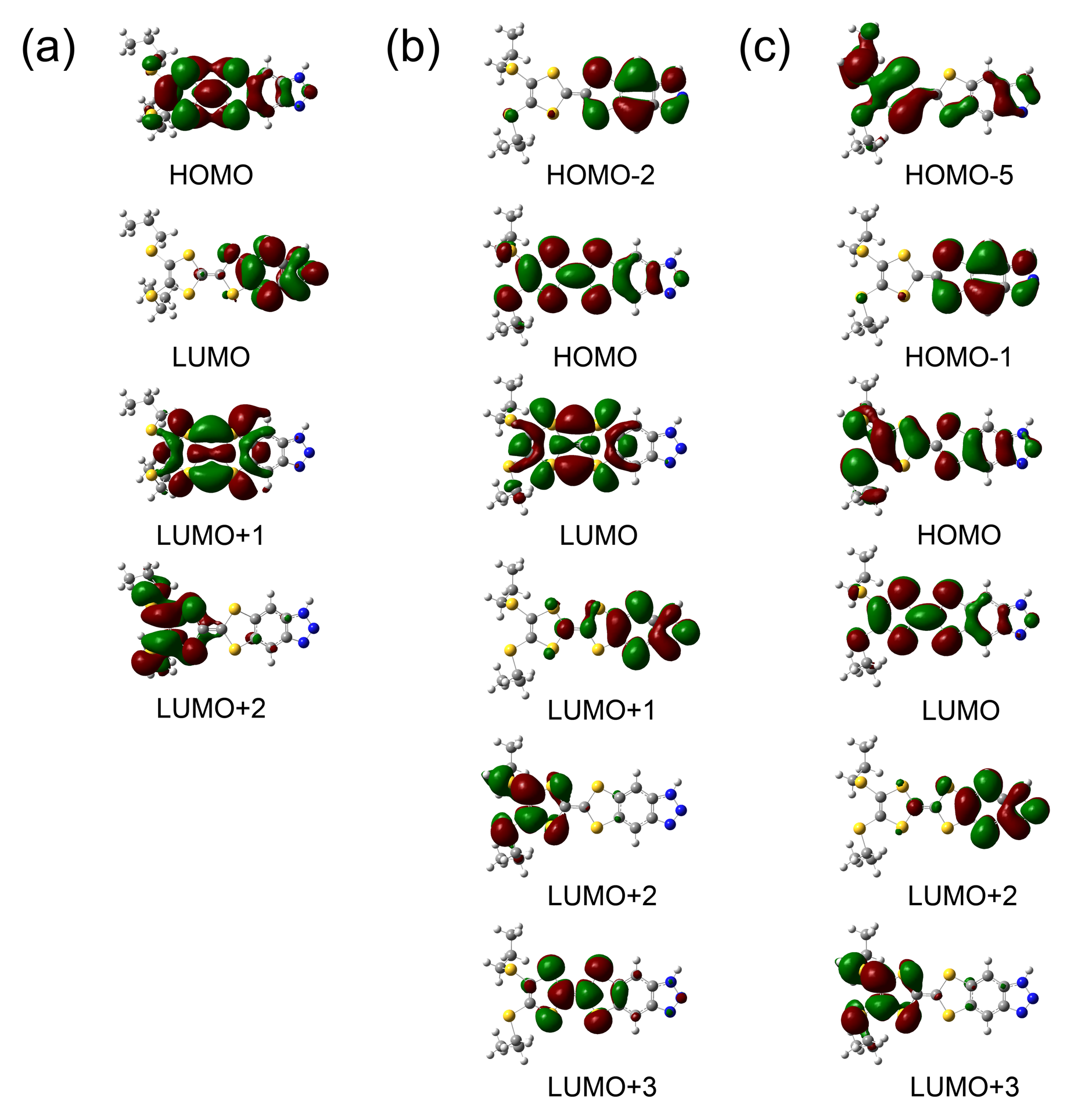


**Figure S8** (a) Frontier molecular orbitals of neutral **L** given in Table S6; (b) Frontier spin-up molecular orbitals and (c) frontier spin-down molecular orbitals of one-electron-oxidized **L** given in Table S6.


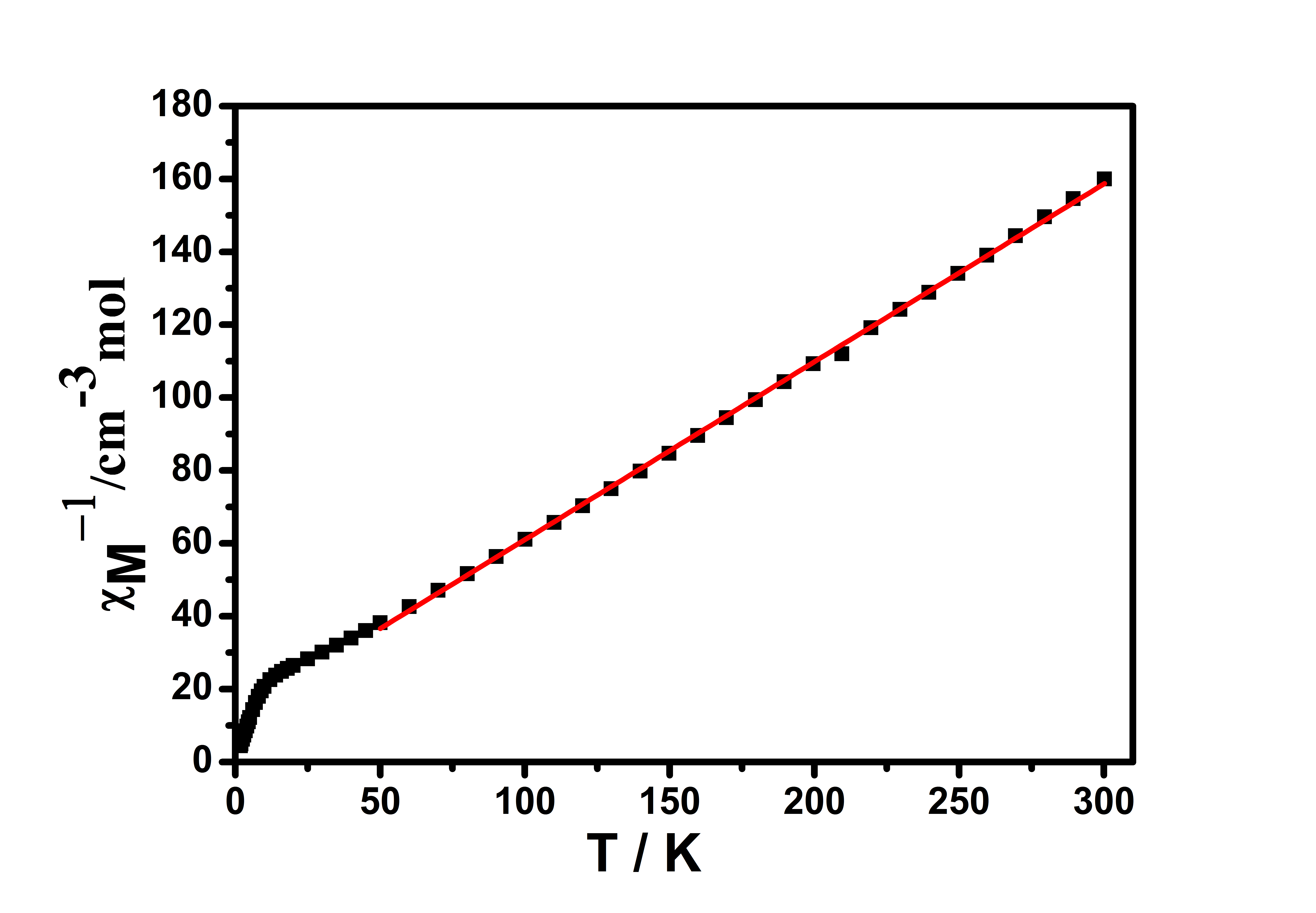


**Figure S9** Plot of 1/χ_M_ vs T for complex **1**. The red solid line is the fitting result by Curie-Weiss law. **
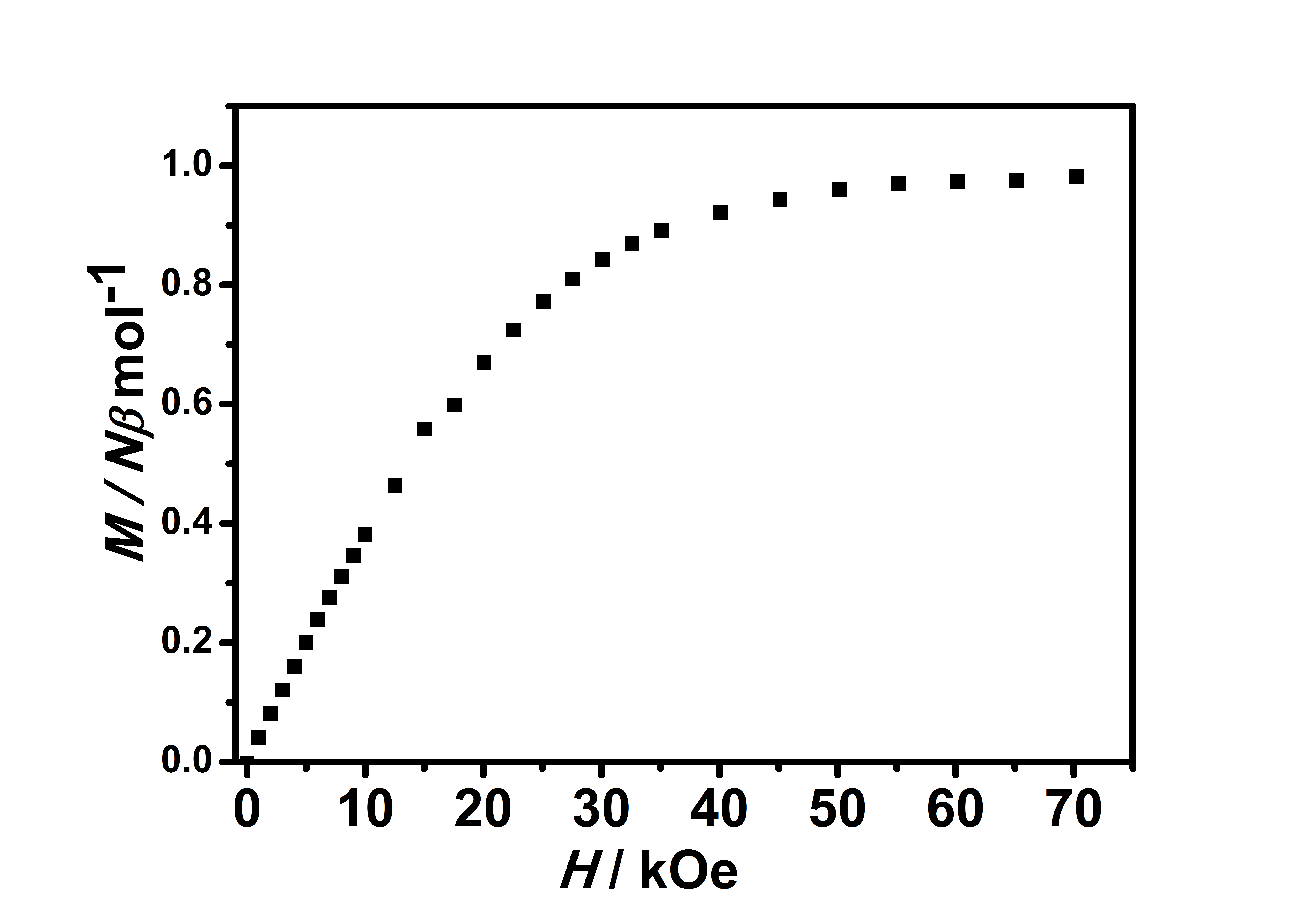
**

**Figure S10** Field dependence of magnetization at 1.8 K.

**Model for calculating the magnetic susceptibilities of 1**

**Figure S11**

The geometry of the Cu5 cluster is highly symmetric, so it is possible to consider only two coupling constans. This geometry gives the energy levels. S_1234_ = algebraic sum of the spins for the outer Cu sites; S_T_ = total algebraic sum of all spins; S_0_ = central spin quantum number; J_1_ = coupling constant between the central and outlying spins; J_2_ = coupling constant between the outlying Cu sites.

S_1_ = S_2_ = S_3_ = S_4_

J_12_ = J_13_ = J_14_ = J_23_ = J_24_ = J_34_ = J_2_

J_01_ = J_02_ = J_03_ = J_04_ = J_1_

E(S_1234_,S_T_) = −*J*_1_S_T_(S_T_ + 1) + *J*_1_S_0_(S_0_ + 1) + (*J*_1_ – *J*_2_)S_1234_(S_1234_ + 1) + 4*J*_2_S(S + 1)

$$\chi_{M}T\text{=}\frac{\text{Ng}^{2}\text{β}^{2}}{3k}\times\frac{\sum_{S_{T}} S_{T}(S_{T}+1)(2S_{T}+1)\exp(-E(S_{T})/kT)}{\sum_{S_{T}} (2S_{T}+1)\exp(-E(S_{T})/kT)}$$

Thus,

$$\chi_{M}T\text{=（0.12505g}^{2}\text{）}\frac{N}{D}$$

Where:

N = 52.5exp([2*J*_1_ + 3*J*_2_]/kT) + 45exp([*J*_1_ – *J*_2_]/kT) + 3exp(–3*J*_2_/kT) + 15exp([–3*J*_1_ + 3*J*_2_]/kT) + 4.5exp([– 2*J*_1_ – *J*_2_]/kT)

D = 6exp([2*J*_1_ + 3*J*_2_]/kT) + 12exp([*J*_1_ – *J*_2_]/kT) + 4exp(–3*J*_2_/kT) + 4exp([–3*J*_1_ + 3*J*_2_]/kT) + 6exp([– 2*J*_1_ – *J*_2_]/kT)

Table S1. Summary of crystallographic data for compounds L and 1.

|  | **L** | **1** |
| --- | --- | --- |
| formula | C_64_H_68_N_12_S_24_ | C_128_H_112_Cu_5_F_12_N_18_O_8_S_40_ |
| *M*_r_ | 1774.74 | 3858.46 |
| cryst system | Triclinic | Monoclinic |
| space group | *P*ī | *C2/c* |
| *a* (Å) | 14.8677(15) | 61.233(3) |
| *b* (Å) | 14.9545(16) | 27.1131(15) |
| *c* (Å) | 19.0414(19) | 38.694(2) |
| *α* (°) | 107.171(2) | 90 |
| *β* (°) | 100.466(2) | 121.013(2)) |
| *γ* (°) | 90.816(2) | 90 |
| *V* (Å^3^) | 3967.3(7) | 55058(5) |
| *Z* | 2 | 8 |
| *ρ*_c_ (g cm^−3^) | 1.486 | 0.931 |
| *T* / K | 296(2) | 291(2) |
| *μ* / mm^−1^ | 0.695 | 3.622 |
| *θ*, deg | 1.43 to 25.50 | 1.68 to 75.00 |
| *F*(000) | 1840 | 15704 |
| index ranges | −18 ≤ h ≤ 17  −17 ≤ k ≤ 18  −21 ≤ l ≤ 23 | −76 ≤ h ≤ 65  0 ≤ k ≤ 33  0 ≤ l ≤ 48 |
| data/restraints/parameters | 14368 / 42 / 935 | 56710 / 0 / 1912 |
| GOF (*F*^2^) | 1.058 | 1.091 |
| *R*_1_^a^, *wR*_2_^b^ (*I* >2*σ*(*I*)) | 0.0941, 0.2041 | 0.0508, 0.1595 |
| *R*_1_^a^, *wR*_2_^b^(all data) | 0.1522, 0.2449 | 0.0601, 0.1643 |

^a^ *R*_1_ = Σ*C* − *F*_c_/Σ*F*_o_. ^b^ *wR*_2_ = [Σ*w*(*F*_o_^2^ − *F*_c_^2^)2/Σ*w*(*F*_o_^2^)]^1/2^

**Table S2. Selected bond lengths (Å) for L**

| Bond Distances (Å) | | | |
| --- | --- | --- | --- |
| C(4)-C(5) | 1.415(7) | C(7)-C(8) | 1.331(8) |
| C(9)-C(13) | 1.349(7) | C(4)-S(2) | 1.739(6) |
| C(5)-S(1) | 1.753(5) | C(7)-S(1) | 1.754(6) |
| C(7)-S(2) | 1.755(5) | C(8)-S(3) | 1.773(5) |
| C(8)-S(4) | 1.757(6) | C(9)-S(4) | 1.764(5) |
| C(13)-S(3) | 1.761(6) | N(1)-N(2) | 1.345(6) |
| N(2)-N(3) | 1.323(7) | C(1)-N(1) | 1.355(7) |

**Table S3. Selected bond lengths (Å) and bond angles (°) for complex 1**

| Bond Distances (Å) | | | |
| --- | --- | --- | --- |
| Cu(1)-N(3) | 1.910(3) | Cu(1)-O(2) | 1.949(3) |
| Cu(1)-N(4) | 1.974(3) | Cu(1)-O(1) | 2.018(2) |
| Cu(1)-N(13) | 2.156(3) | Cu(2)-N(6) | 1.882(3) |
| Cu(2)-O(4) | 1.898(3) | Cu(2)-N(16) | 1.954(3) |
| Cu(2)-O(3) | 2.039(2) | Cu(2)-N(7) | 2.195(3) |
| Cu(3)-O(5) | 1.904(2) | Cu(3)-O(6) | 1.936(3) |
| Cu(3)-N(9) | 2.030(3) | Cu(3)-N(10) | 2.068(3) |
| Cu(3)-N(1) | 2.136(3) | Cu(4)-N(17) | 1.920(3) |
| Cu(4)-N(8) | 2.008(3) | Cu(4)-N(14) | 2.034(3) |
| Cu(4)-N(2) | 2.099(3) | Cu(4)-N(5) | 2.284(3) |
| Cu(4)-N(11) | 2.302(3) | Cu(5)-O(7) | 1.921(3) |
| Cu(5)-N(15) | 1.922(3) | Cu(5)-N(12) | 1.960(3) |
| Cu(5)-O(8) | 1.979(3) | Cu(5)-N(18) | 2.163(3) |
| Bond angles (°) | | | |
| N(3)-Cu(1)-O(2) | 167.80(12) | N(3)-Cu(1)-N(4) | 92.64(13) |
| O(2)-Cu(1)-N(4) | 86.95(11) | N(3)-Cu(1)-O(1) | 85.91(12) |
| O(2)-Cu(1)-O(1) | 91.32(10) | N(4)-Cu(1)-O(1) | 164.94(12) |
| N(3)-Cu(1)-N(13) | 93.92(12) | O(2)-Cu(1)-N(13) | 98.16(11) |
| N(4)-Cu(1)-N(13) | 100.03(12) | O(1)-Cu(1)-N(13) | 95.02(11) |
| N(17)-Cu(4)-N(8) | 90.56(12) | N(17)-Cu(4)-N(14) | 87.28(13) |
| N(8)-Cu(4)-N(14) | 171.05(12) | N(17)-Cu(4)-N(2) | 174.61(12) |
| N(8)-Cu(4)-N(2) | 93.43(11) | N(14)-Cu(4)-N(2) | 89.33(12) |
| N(17)-Cu(4)-N(5) | 87.13(12) | N(8)-Cu(4)-N(5) | 93.45(11) |
| N(14)-Cu(4)-N(5) | 95.12(11) | N(2)-Cu(4)-N(5) | 89.01(12) |
| N(17)-Cu(4)-N(11) | 91.64(12) | N(8)-Cu(4)-N(11) | 85.40(11) |
| N(14)-Cu(4)-N(11) | 85.98(11) | N(2)-Cu(4)-N(11) | 92.30(12) |

**Table S4.** Summary of cyclic voltammetric data on **L** and **1** collected at 100 mV/s in 0.1 M [*n*-Bu_4_N]PF_6_/DCM.

| Compound | Couple | Δ*E_p_* (mV) | *E_1/2_* (V) | *i_pa_* (μA) | *i_pc_* (μA) | *i_pa_/i_pc_* | *i_pc_/i_pa_* |
| --- | --- | --- | --- | --- | --- | --- | --- |
| **L** | TTF^•+^/TTF | 81 | 0.175 | 1.230 | 0.985 | 1.25 | - |
|  | TTF^2+^/TTF^•+^ | 81 | 0.575 | 1.206 | 1.023 | 1.18 | - |
| **1** | TTF^•+^/TTF | 59 | 0.176 | 1.340 | 1.171 | 1.14 | - |
|  | TTF^2+^/TTF^•+^ | 105 | 0.592 | 1.010 | 0.960 | 1.05 | - |
|  | Cu^2+^/Cu^+^ | 197^†^ | -1.360 | 0.272 | 0.534 | - | 1.96 |

^†^These values are approximates only due to the quasi-reversible nature of these processes.

**Table S5.** Main calculated optical transitions for neutral and one-electron-oxidized ligand **L**.

| Major contributes | Oscillator strength | λ/nm(calcd) | λ/nm(exptl) |
| --- | --- | --- | --- |
| **L** | | | |
| HOMO→LUMO | 0.0857 | 423 |  |
| HOMO→LUMO+1 |  |  |  |
| HOMO→LUMO | 0.0833 | 412 | 381 |
| HOMO→LUMO+1 |  |  |  |
| HOMO→LUMO+2 | 0.055 | 360 |  |
| **L^·+^** | | | |
| HOMO(β)→LUMO(β) | 0.2633 | 803 | 800 |
| HOMO-1(β)→LUMO(β) | 0.0106 | 656 |  |
| HOMO(β)→LUMO(β) | 0.0381 | 483 | 530 |
| HOMO(α)→LUMO(α) |  |  |  |
| HOMO(α)→LUMO+2(α) | 0.0415 | 422 | 460 |
| HOMO(β)→LUMO+3(β) |  |  |  |
| HOMO(α)→LUMO+1(α) | 0.2501 | 416 | 430 |
| HOMO-2(α)→LUMO+1(α) | 0.0151 | 403 |  |
| HOMO-1(β)→LUMO+2(β) |  |  |  |
| HOMO-5(β)→LUMO(β) | 0.0333 | 392 |  |
| HOMO(α)→LUMO+3(α) | 0.1819 | 380 |  |

**References**

1. Guégano, X. *et al*. **P**ronounced electrochemical amphotericity of a fused donor–acceptor compound: a planar merge of TTF with a TCNQ-type bithienoquinoxalin. *Chem.-Eur. J.* **15**, 63-66 (2009).

2. *SAINT-Plus*, version 6.02; Bruker Analytical X-ray System: Madison, WI, 1999.

3. Sheldrick, G. M. *SADABS* *an empirical absorption correction program*; Bruker Analytical X-ray Systems: Madison, WI, 1996.

4. Sheldrick, G. M. *SHELXTL-97*; Universität of Göttingen: Göttingen, Germany, 1997.
